# FastViFi: Fast and accurate detection of (Hybrid) Viral DNA and RNA

**DOI:** 10.1093/nargab/lqac032

**Published:** 2022-04-26

**Authors:** Sara Javadzadeh, Utkrisht Rajkumar, Nam Nguyen, Shahab Sarmashghi, Jens Luebeck, Jingbo Shang, Vineet Bafna

**Affiliations:** Department of Computer Science & Engineering, UC San Diego, La Jolla, California, USA; Department of Computer Science & Engineering, UC San Diego, La Jolla, California, USA; Boundless Bio, Inc. 11099 N Torrey Pines Rd, La Jolla, CA, USA; Department of Electrical and Computer Engineering, UC San Diego, La Jolla, California, USA; Bioinformatics & Systems Biology Graduate Program, UC San Diego, La Jolla, California, USA; Department of Computer Science & Engineering, UC San Diego, La Jolla, California, USA; Department of Computer Science & Engineering, UC San Diego, La Jolla, California, USA; Boundless Bio, Inc. 11099 N Torrey Pines Rd, La Jolla, CA, USA; Moores Cancer Center, UC San Diego, La Jolla, California, USA

## Abstract

DNA viruses are important infectious agents known to mediate a large number of human diseases, including cancer. Viral integration into the host genome and the formation of hybrid transcripts are also associated with increased pathogenicity. The high variability of viral genomes, however requires the use of sensitive ensemble hidden Markov models that add to the computational complexity, often requiring > 40 CPU-hours per sample. Here, we describe FastViFi, a fast 2-stage filtering method that reduces the computational burden. On simulated and cancer genomic data, FastViFi improved the running time by 2 orders of magnitude with comparable accuracy on challenging data sets. Recently published methods have focused on identification of location of viral integration into the human host genome using local assembly, but do not extend to RNA. To identify human viral hybrid transcripts, we additionally developed ensemble Hidden Markov Models for the Epstein Barr virus (EBV) to add to the models for Hepatitis B (HBV), Hepatitis C (HCV) viruses and the Human Papillomavirus (HPV), and used FastViFi to query RNA-seq data from Gastric cancer (EBV) and liver cancer (HBV/HCV). FastViFi ran in <10 minutes per sample and identified multiple hybrids that fuse viral and human genes suggesting new mechanisms for oncoviral pathogenicity. FastViFi is available at https://github.com/sara-javadzadeh/FastViFi.

## INTRODUCTION

DNA viruses are important infectious agents mediating a large number of human diseases, cancer in particular. These include human papillomaviruses (HPV), Epstein-Barr Virus (EBV), Hepatitis B and C viruses (HBV/HCV) and other viruses involved in the etiology of various cancers (1). For example, HPV (mainly HPV-16 and HPV-18) is reported to cause at least 5% of all cancers globally (2), with high risk HPV reported as the causal agent for almost all cervical cancers (3–5). Similarly, oropharynx cancer, often driven by human papillomavirus (HPV) type 16 (6), is now the second-fastest growing cause of cancer death and the third-fastest growing in frequency among solid organ cancers in the U.S. according to https://progressreport.cancer.gov/diagnosis/incidence (2020). EBV (HHV-4), known as the cause of mononucleosis, is also associated with various non-malignant, premalignant, and malignant lymphoproliferative diseases including Burkitt lymphoma, autoimmune diseases (e.g. rheumatoid arthritis), Sjögren’s syndrome, and multiple sclerosis. In 2010, nearly 200,000 global cancer cases per year were attributable to EBV (7), and the rate of infections is continually growing.

Analytic pipelines have been developed to identify viral sequences from Illumina NGS data (Verse (8), VirusFinder (9), ViralFusionSeq (10), and Virus-Clip (11)). Despite these advances, there is tremendous variability even between strains of the same viral subtype, and we cannot preclude the possibility that novel viral strains are missed in sequence based searches. Hirose et. al. (12) found an average of 4.6 per 100bp nucleotide substitutions within HPV-16 genomes in each host. Therefore, identifying the infectious viral strain remains challenging.

Analyses of viral mediated cancer data has associated HPV integration into the host genome with increased DNA instability (12), hybrid virus-human extrachromosomal DNA formation (4,13,14) with dramatically increased expression of hybrid viral-human transcripts (15). The presence of hybrid HPV-human transcripts encoding the viral oncogene E6 are associated with poor pathogenesis (16). The difficulty of discovering of hybrid host-viral reads (either DNA or RNA) is tightly related to the difficulty of discovering viral reads as the host (e.g human) sequence is not highly mutated and easy to identify. However, the discovery of a viral-human hybrid read does not immediately yield the location of integration into the host genome because the host read may be drawn from a repetitive region such as Alu, LINE, or ribosomal RNA genes.

New methods like VIRUSBreakend (17) focus specifically on improved detection of viral integration locations. VIRUSBreakend uses a keyword matching approach for the detection of viruses, followed by a local genome assembly that allows it to detect integration even in low-complexity regions of the host genome that are missed by other methods. Because of its requirement of local assembly (suitable for DNA, but not RNA), we were unable to systematically test VIRUSBreakend for identification of diverged viral reads, or for detection of host-viral hybrid transcripts using RNA-seq samples (see Results).

From a different side, deep learning tools such as DeepVirFinder (18) have reported excellent results on identifying viral sequences drawn from larger viral families. However, when training in a host-viral setting, the networks often end up learning what is ‘not-host,’ which is an easier problem when the host has well characterized and conserved sequence (e.g. human). Unfortunately, in realistic host-viral settings, the sampled data often includes bacterial and fungal sequences (19,20), which are not known *a priori* and are therefore not included in the training set. We tested DeepVirFinder in these ‘open-set’ scenarios and observed a significant degradation in performance (Results). We had previously developed ViFi which used an ensemble of Hidden Markov Models (HMMs) to identify highly variable strains with high accuracy (15). However, ViFi is slow, requiring (for example) 52 cpu-hours to identify HPV from human cancer whole genome sequenced samples with 352 million reads, and is outperformed by VIRUSBreakend in detecting sites of integration.

Here, we describe Filter-associated-ViFi (*FastViFi*), which uses keyword based filtering to speed up ViFi without losing sensitivity, and focuses specifically on detection of (diverged) viral reads and hybrid host-viral reads. We benchmarked FastViFi on a collection of simulated and tumor whole genome data to measure speed, precision, and recall, and compared our results to DeepVirFinder, Kraken (21) (a k-mer matching method) and ViFi. we tested the importance of having two Kraken filters instead of a single Kraken filter by comparing the performance of single-stage vs two-stage Kraken filtering. As VIRUSBreakend (17) is focused on integration sites and does not work for sparse, viral-only read data, we compared partially against it for host-viral integration and identification of hybrid viral-human transcripts in RNA-seq data. Finally, we tested the relevance of FastViFi results on three TCGA cancer datasets with Human Papilloma virus, Hepatitis B virus, Hepatitis C virus, and Epstein Barr virus infections, focusing specifically on hybrid human viral RNA detection.

The results suggest that FastViFi can accurately identify diverged viral strains, and host-viral hybrid transcripts. Our results also identified possible novel mechanisms of cancer pathology due to hybrid transcript formation in HBV and EBV mediated cancers.

## MATERIAL AND METHODS

FastViFi relies on a filtering strategy to increase speed while maintaining high sensitivity. The filter aims to rapidly discard a large majority of the (mostly human) reads, while retaining most of the true viral or viral-human hybrid reads. The small number of filtered reads are subsequently analyzed and confirmed using a slower but more accurate method (ViFi). We characterize the filter by (a) speed and (b) its sensitivity (or *recall*) in retaining true viral reads. However, *precision*, defined as the fraction of filtered reads that are truly viral, is not a major concern because the filtered reads are subsequently confirmed using ViFi. Instead, we characterize the filter by its *efficiency*–defined as the ratio of the number of query reads to the number of filtered reads. High efficiency of the filters decreases the running time of the overall computation as the slower but accurate ViFi is deployed only on the filtered reads.

**Figure 1.**
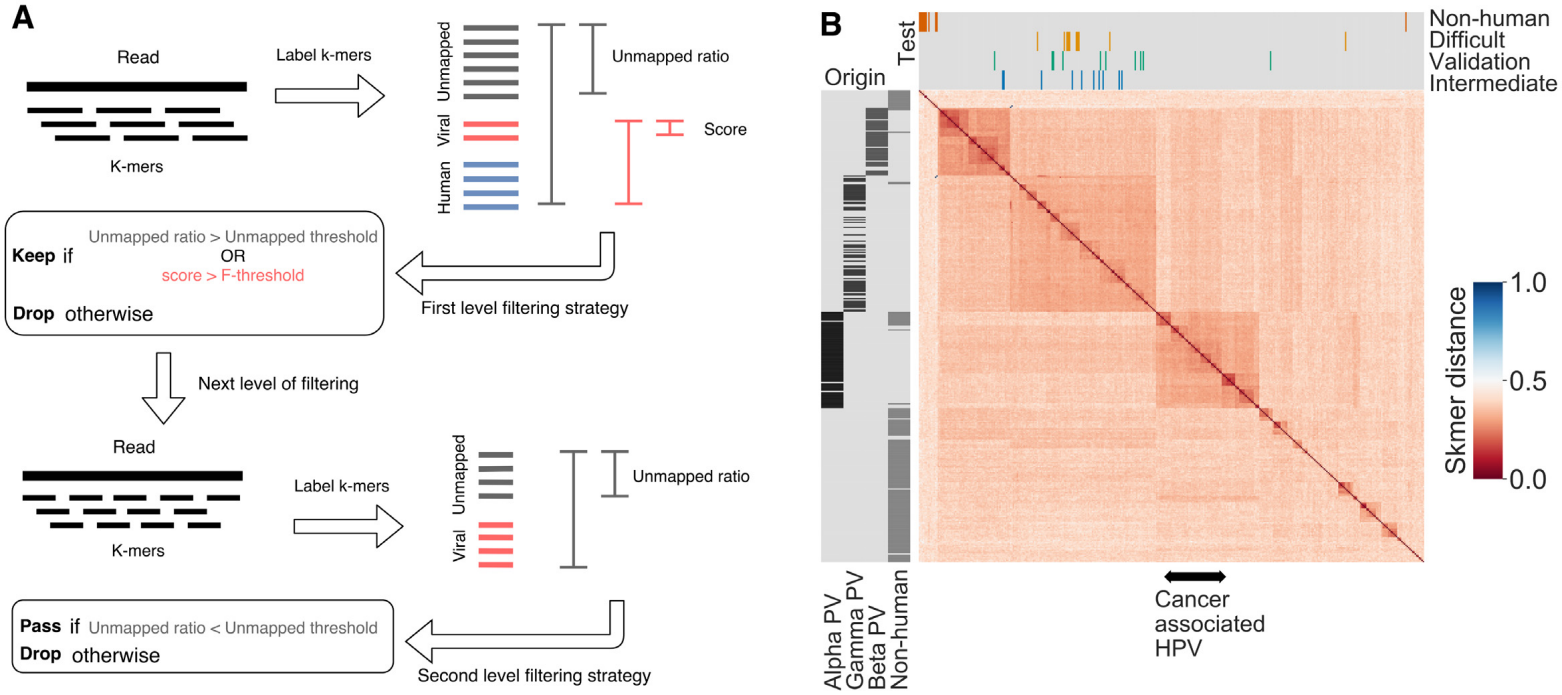
**Panel A** Pipeline describing the FastViFi filter strategy. The viral, human and unmapped k-mers are used to compute the unmapped ratio and score used for filtering. **Panel B** Pairwise Skmer distance between reference papillomavirus strains computed using Skmer(22)(k=12), followed by hierarchical clustering. Known PV families Alpha, Beta and Gamma are labeled along with the marks for the test data sets–HPV-intermediate, HPV-validation, HPV-difficult and PV-non-human. Cancer associated HPV strains HPV16,18,6,11,31,33, 45, 52, and 58 cluster together in the alpha PV class.

The pipeline implementing the filtering and matching strategy, called *FastViFi*, is shown in Figure 1A and described in this section. Broadly, for parameters *k*, *t*, *u*, we *filtered* a read if at least *uk*-mers matched a human and virus reference database, and the fraction of *k*-mers that match virus exceeded *t*. The problem remained challenging for viruses because of their high variability. Large *k*-mers tended to lose sensitivity while smaller values of *k* lowered the efficiency. Therefore, we deployed a two stage filter with the first filter aimed towards removing human reads from the sample, and the second filter aimed at enriching for viral reads. We additionally developed a method called *FastViFiAnalytics* to automatically estimate sensitivity and efficiency of the two-stage filters for any choice of parameters, and enable the users to optimize the method for their needs.

Finally, we extended the range of oncoviral families supported by developing new Hidden Markov models for Human Papilloma Virus (95 models), Hepatitis B (19), Hepatitis C (27) Virus and Epstein Barr Virus (5 models); we improved the software to allow for the incorporation of other viral families, for sub-family classification, and to provide more flexible controls for a range of sensitivity, and run time requirements.

### The FastViFi method

Formally, consider a data set of sequences *D*. The objective is to identify a subset *V*(*D*)⊆*D* of viral sequences. Define a *filterF* as a procedure that takes a data set *D* and returns a subset *F*(*D*)⊆*D*. A filter is characterized by its (a) *running-time* denoted by *T*_*F*_(*D*); (b) *efficiency* defined as

$$\begin{equation*} \text{efficiency} = E = \frac{\vert D\vert }{\vert F(D)\vert }\; ; \end{equation*}$$

and, (c) *sensitivity or recall*, defined by

$$\begin{equation*} \text{recall} = \frac{\vert F(D)\cap V(D)\vert }{\vert V(D) \vert } \end{equation*}$$

A desired filter is fast, efficient and sensitive. Using this definition, ViFi can be thought of as a filter that outputs *V*(*D*)⊆*D* with very high efficiency and recall but slow running-time *T*_*V*_. We attached a filter with high speed *T*_1_, efficiency *E*_1_ and high recall and ran ViFi on the filtered output to improve overall performance. The new overall running time would be *T*_1_(*D*) + *T*_*V*_(|*D*|/*E*_1_), and governed by the speed and efficiency of the attached filter. Also, the $\text{recall}=\frac{\vert F(D)\cap V(D)\vert }{\vert V(D) \vert }$ depends critically on the recall of *F*. Filters can be composed, so that a filter is deployed on the filtered output of a previous filter. We selected filters by optimizing an efficiency versus recall trade-off.

#### Keyword match filters

We selected Kraken (21) as the tool to estimate the k-mer composition of the viral reads. For the parameter *k*, a taxonomy tree, and a set of reference sequences, Kraken builds an index based on k-mer length *k* from the references to rapidly annotate query reads. Specifically, Kraken reported the detected taxonomy label for each k-mer in a query read. For k-mers that mapped to multiple nodes in the taxonomy tree, Kraken reported the lowest common ancestor. Finally, the k-mers that did not map to any node in the taxonomy tree were reported as unmapped.

#### Kraken references

To build custom Kraken indices, human and viral references should be provided. For human reference, we used the setting in Kraken to download the reference from the NCBI library. For viral strains, we provided the reference FASTA files identical to the references in ViFi. We augmented the taxonomy tree with all viruses in the reference as direct descendants of the Viruses (Rank:superkingdom) node wit taxid:10239.

#### Level-1 filtering

FastViFi is composed of two filters, with the goals of discarding human reads first, followed by discarding unknown contaminant reads while retaining target viral reads later in the second filter. Each filter had 3 parameters denoted by *k*_1_, *u*_1_, *t*_1_, and *k*_2_, *u*_2_, *t*_2_, respectively.

For read *r*, parameter *k*_1_ let *n*_$v$_(*r*, *k*_1_) (respectively, *n*_*h*_(*r*, *k*_1_)) denote the number of k-mers labeled as viral (respectively, human). We defined a v-score for *r* as:

$$\begin{equation*} \text{v-score}(r,k_1) = \frac{n_{v}(r,k_1)}{n_{v}(r,k_1) + n_{h}(r,k_1)} \end{equation*}$$

A read was classified by the Level-1 filter as *non-human* (and retained for the next filter)if and only if

$$\begin{equation*} \text{v-score}(r,k_1)\ge t_1 \end{equation*}$$

or

$$\begin{equation*} \frac{n_u(r,k_1)}{n_u(r,k_1)+n_v(r,k_1)+n_h(r,k_1)}\ge u_1 \end{equation*}$$

A paired-end read passed this filter if at least one of the paired-ends passed the filter.

#### Level-2 filtering

The level-2 filter used the same criteria as level-1 but the parameters *k*_2_, *t*_2_ were chosen to accept viral sequences and discard contaminants. Specifically, only the viral k-mer indexes in Kraken reference were chosen for mapping k-mers.

#### FastViFi filter parameter grid-search

We performed a grid-search on *k*_1_, *k*_2_, *u*_1_, *t*_1_ and *t*_2_ as an experimental optimization of the parameters for the Level-1 and Level-2 composed or FastViFi filters. The grid search on the FastViFifilter is performed on the following parameters and values: *k*_1_ ∈ {20, 25, 30}, *u*_1_ ∈ {0.2, 0.4, 0.6, 0.8, 0.9, 1.0}, *t*_1_ ∈ {0.1, 0.2, 0.3, 0.4, 0.5, 0.6, 0.7, 0.8}, *k*_2_ ∈ {14, 18, 22, 24, 26, 28}, *t*_2_ ∈ {0.2, 0.4, 0.6, 0.8, 0.9, 1.0}.

#### FastViFi configurations

FastViFi is configured to have either high read level sensitivity, where the goal is to maximize the viral reads detected, or sample level sensitivity, where the goal is to rapidly label samples as positive or negative for a viral genome. The parameters for each configuration were extracted from the grid search values. The proposed way to run FastViFi on a dataset with a large number of samples is to first run FastViFi on *sample-level* configuration. Then, on the samples where viral reads were detected, run FastViFi on *read-level*.

The *sample-level* configuration was set based on the experiment resulting the maximum combination of recall and efficiency where recall was at least 0.50 and efficiency was at least 830. Specifically, *k*_1_ = 25, *k*_2_ = 22, *t*_1_ = 0.4, *u*_1_ = 0.8 · *n*(*r*, *k*_1_), *t*_2_ = 0.1 · *n*(*r*, *k*_2_). Similarly, the *read-level* configuration was set based on the experiment resulting in the maximum efficiency when recall was at least 80%, resulting in a choice of *k*_1_ = 25, *k*_2_ = 18, *t*_1_ = 0.8, *u*_1_ = 0.6 · *n*(*r*, *k*_1_), *t*_2_ = 0.2 · *n*(*r*, *k*_2_). The software distribution includes scripts to find configurations with any desired minimum recall and efficiency based on experiments on the HPV-validation dataset.

#### FastViFi input

The input to FastViFi could be either a pair of FASTQ files containing sequenced paired end reads, or a BAM file containing those reads aligned to the human genome. For a given BAM file containing paired end sequenced reads aligned to the human genome, an additional *alignment-based* filter (script available in the repository) discards paired end reads where both mates are mapped to the human genome. The remaining reads are stored as FASTQ files and processed by FastViFi.

#### FastViFi output

FastViFi reports the paired-end reads where both mapped to viral references, or where one mate is mapped to the human genome and the other is either mapped to a viral reference or a reference HMM, as well the hybrid DNA/RNA loci and the supporting reads.

#### Viral HMMs in ViFi

We created viral references for HPV, HBV, HCV and EBV based on 337, 73, 111, and 23 strains respectively. The number of FastViFi HMMs created for HPV, HBV, HCV and EBV are 95, 19, 27, and 5 HMMs respectively.

#### Modifications in Kraken2

In the modified version of Kraken2 used in the FastViFi pipeline, the computations on score and thresholds illustrated in Figure 1 are embedded in the Kraken2 process, avoiding an extra filtering step on Kraken2 output files. The filtering strategy is configurable conveniently by input flags to the modified Kraken2 software. Based on the input threshold values, as soon as a decision on discarding a read is made, further processes on the read is interrupted.

#### Modifications in ViFi

ViFi primarily utilizes HMM alignment to target hybrid DNA or hybrid RNA for variant viral strains. Specifically, the original ViFi software aligns paired end reads to HMMs if one mate is mapped to the human genome and one is unmapped. To broaden the scope of HMM alignment, we modified ViFi to align paired end reads to HMMs if at least one mate is unmapped. This modification enables us to extract variant viral strains in cases with no hybrid DNA or hybrid RNA. Moreover, our modifications in the read-level configuration avoids considering a BWA alignment to the human or viral genomes a valid alignment in case a significant number (80%) of read bases are soft-clipped. Additionally, we added a feature to report further details on HMM alignment such as alignment location on HMM and the corresponding set of viral references constructing the HMM.

### FastViFiAnalytics for selection of parameters for 2-level filtering.

Let ℓ denote the number of k-mers in a query sequence, and *n* denote the number of k-mers in a data set. Let *d* be the genomic distance of the query sequence from its nearest ortholog in the data set. The expected number of k-mer matches between the query and and the data set is given by

(1) $$\begin{equation*} \mathbb {E}[{\rm \# k-mer matches}] =\lambda _v =\ell (1-d)^k \end{equation*}$$

The value of *d* depends upon context. For viral query and viral data sets, we use *d* ∈ {0.05, 0.1, 0.15, 0.2} depending upon the hardness of the data set (See data set construction below). Similarly, for a human (host) query is mapped to a human (host) reference, *d*_*h*_ = 0.01 (largely due to sequencing errors), and

$$\begin{equation*} \lambda _h = \ell (1-d_h)^k \end{equation*}$$

Finally, consider the case of a query that is not from the same group as the index, and any k-mer match is just by chance. Let *d*_*r*_ denote its genomic distance from the closest sequence in the index. Here *d*_*r*_ is very high (we choose *d*_*r*_ = 0.75) but the k-mer may match anywhere in the reference containing *n* k-mers. Therefore,

$$\begin{equation*} \mathbb {E}[{{\rm \# k-mer matches to query}}]=\lambda _{r,n}=\ell n (1-d_r)^k \end{equation*}$$

As the probability of a single k-mer matching is small, we assume in each case that the number of matches *u* are Poisson distributed with the parameter λ conditioned on there being at most ℓ matches. Thus, the probability of matching at most *t* of the ℓ k-mers is approximated to

$$\begin{equation*} \frac{\sum _{u=0}^{t}\text{Poisson}(\lambda ,u)}{\sum _{u=0}^{\ell }\text{Poisson}(\lambda ,u)} \end{equation*}$$

#### Level-1 filtering

The goal of level-1 filtering is to remove all host reads. Specifically, for parameters *k*_1_, *u*_1_, *t*_1_, we *retain* a read only if *no more* than *u*_1_ℓ *k*_1_-mers are matched (i.e. non-host) OR if *t*_1_ℓ or higher number *k*_1_-mers match to virus. The second filtering condition ensures that viral reads are retained, providing an estimate of sensitivity as:

(2) $$\begin{equation*} \text{Sensitivity}_1 \simeq \frac{\sum _{u=t_1\ell }^{\ell }\text{Poisson}(\lambda _v,u)}{\sum _{u=0}^{\ell }\text{Poisson}(\lambda _v,u)} \end{equation*}$$

The probability of retaining (not discarding) a host sequence is approximated by

$$\begin{equation*} P_h\simeq \frac{\sum _{u=0}^{u_1\ell } \text{Poisson}(\lambda _h,u)}{\sum _{u=0}^{\ell } \text{Poisson}(\lambda _h,u)} \end{equation*}$$

Define *P*_*r*_ as the prior probability of a sequence being a contaminant sequence in the sample. We estimate *P*_*r*_ ≃ 0.05 , *d*_*r*_ = 0.75. Let *n* = 3 × 10^9^ and *n*_$v$_ = 337 × 7500 represent the number of k-mers in the human genome and viral references respectively.

$$\begin{eqnarray*} \text{Pr[random-match]} &\simeq& \frac{\sum _{u=0}^{t_1\ell _1} \text{Poisson}(\lambda _{r, n_h},u)}{\sum _{u=0}^{\ell _1} \text{Poisson}(\lambda _{r, n_h},u)}\\ \text{Pr[pass-filter]} &=& (1 - P_r) \cdot P_h + P_r \cdot \text{Pr[random-match]} \\ \text{Efficiency}_1 &\simeq& \frac{1}{\Pr [\text{pass-filter}]} \end{eqnarray*}$$

In the efficiency calculations for the first level of filtering, we only count for the human reads and contaminant reads, ignoring the viral reads as the number of viral reads are negligible when compared to contaminant and human reads.

#### Level 2

In the second level filtering (parameters *k*_2_, *t*_2_, we search only against the viral reference and retain a read if at least *t*_2_ℓ of the *k*_2_-mers match. The probability of retaining a read is related to the prior probability *P*_$v$_ of being a viral sequence, and the probability of a chance match to a non-viral sequence.

(3) $$\begin{equation*} \Pr [\text{match}] =(1-P_v) \frac{\sum _{u=t_2\ell }^{\ell } \text{Poisson}(\lambda _{r,n},u)}{\sum _{u=0}^{\ell } \text{Poisson}(\lambda _{r,n},u)} \end{equation*}$$

(4) $$\begin{equation*} + P_v\frac{\sum _{u=t_2\ell }^{\ell } \text{Poisson}(\lambda _v,u)}{\sum _{u=0}^{\ell } \text{Poisson}(\lambda _v,u)} \end{equation*}$$

(5) $$\begin{equation*} \text{Efficiency}_2 =\frac{1}{\Pr [\text{match}]} \end{equation*}$$

(6) $$\begin{equation*} \text{Sensitivity}_2 = \frac{\sum _{u=t_2\ell }^{\ell } \text{Poisson}(\lambda _v,u)}{\sum _{u=0}^{\ell } \text{Poisson}(\lambda _v,u)} \end{equation*}$$

The overall efficiency and sensitivity of the composite filter is the product of the efficiencies and sensitivities of the two filters, respectively.

### Using DeepVirFinder

We compared FastViFi against DeepVirFinder (18) (DVF), the deep-learning gold standard for HPV detection. DVF which uses a CNN-based discriminative classifier to classify human and HPV sequences (18).

We re-trained DeepVirFinder using our training data HPV-ref and reads from the human genome. We optimized the model using 500 filters in the convolutional layer, and 100 nodes in the dense layer. We trained for the model until convergence using 8 GeForce GTX 1080 Ti GPUs. As recommended by the software, if the loss on the validation set did not improve for 7 epochs (the ‘patience’ time), we halted the training.

### Benchmark data sets

We collected Human Papillomavirus (HPV) references from the Papillomavirus Episteme dataset (PaVE). PaVE portal is accessible through https://pave.niaid.nih.gov. We used all 337 HPV strains recorded prior to October 2017 as the *training/reference* data and refer to this dataset as **HPV − ref**. All sequences recorded after this date were referred to as *novel*

We used Skmer (22) to compute the minimum distance of every novel sequence to any sequence in HPV-ref. Supplementary Figure S4 shows the distribution of computed Skmer distances of each novel sequence with respect to HPV-ref, with the distance ≥0.1. We chose the four test data sets based on first (**HPV-intermediate**), 33^*rd*^ (**HPV-Validation**), 66^*th*^ (**HPV-difficult**) and last (**PV-non-human**) percentile of the distribution. Supplementary Table S11 describes the strain names for each of the test data sets.

Each test data set consisted of simulated reads (using ART (23)) from the selected viral genomes, the human genome (GRCh38 assembly), and contaminant genomes (bacterial and fungal) (19) as follows:

- 100k paired-end human originated reads simulated from the human genome (GRCh38 assembly) with coverage of 0.01.
- Around 240 paired-end virus originated reads simulated from 10 viral strains. For each viral genome, reads were simulated with coverage of 1. The exact number for each dataset varied depending on the length of the viral genomes.
- 5250 paired-end reads sampled from five bacterial and three fungal strains (Supplementary Table S12) chosen randomly from the NCBI taxonomy tree. Reads were simulated from bacterial and fungal genomes and sub-sampled randomly, resulting in 2625 paired-end bacterial reads and 2625 paired-end fungal reads.

All simulated data sets include the same human and contaminant reads, i.e., only the viral reads are updated for each test data set, and percentage of human to viral to contaminant reads was 100,000:240:5,250.

### FastViFi analysis

#### Using FastViFi for identifying DNA integration and hybrid transcripts

Hybrid DNA integration and hybrid transcripts are reported after clustering reads within the same junction and reporting the number of distinct junctions. Reads are considered to be within the same junction if they belong to the same sample i.e., same individual, the human part of the hybrid reads are within 300bp of each other, and the viral part of the hybrid reads are within 100bp of each other.

#### Hybrid transcript expression

We collected normalized RNA expression values for our HBV samples and EBV samples from the HCC and STAD studies respectively, from TCGA. We computed statistics for change in gene expression for specific genes with recurrent hybrid transcripts. For each gene of interest, we refer to samples carrying hybrid transcripts for that gene as positive; samples were labeled negative for that gene otherwise. We computed the r-statistic as the ratio of the median expression of the positive and negative samples. The Wilcoxon Rank Sum test is often used to estimate the significance of the change in gene expression values, but was not used due to the small number of positive samples. Instead, we performed a rank-permutation test as follows. For each gene, we computed the rank-sum statistic *s* as the sum of the global ranks of the gene in the positive samples. We ran 10,000 trials with random permutations of the positive/negative status and computed the p-value as the fraction of trials in which the permuted rank-sum statistic was more extreme than *s*.

### Software availability

FastViFi is available at https://github.com/sara-javadzadeh/FastViFi.

We modified Kraken and ViFi tools to work more efficiently in FastViFi pipeline. The forked repositories are available at

https://github.com/sara-javadzadeh/kraken2 and

https://github.com/sara-javadzadeh/ViFi.

## RESULTS

### Training data

We collected Human Papillomavirus (HPV) references from the Papillomavirus Episteme data set (PaVE). We chose all 337 HPV strains discovered prior to October 2017 as the *training/reference* data and refer to this data set as **HPV − ref**.

### Estimating complexity/divergence of viral data

We used Skmer (22) to estimate the distance between HPV genomes based on the number of shared k-mers (*k* = 12) followed by hierarchical distance based clustering (Figure 1B). Notably, the known Papilloma virus (PV) classes Alpha, Beta, and Gamma (derived by querying PaVE portal), each formed their own larger clusters, while the non-human PV genomes were scattered into smaller clusters or singletons.

### Test data

To generate realistic scenarios, we separated the training and test data by date. Specifically, training data was comprised of papilloma viral (PV) strains available on the PaVE platform earlier than October 2017. Viruses submitted later than the date were reserved for testing. We separated sequences by divergence based on their closest distance to any viral genome in the training set and created 4 data sets of increasing divergence (Material and Methods). Among these four, an **HPV-validation** data set was used in a feedback loop to fine-tune the parameters learned from HPV-ref in FastViFi. Three other data sets of increasing complexity–**HPV-intermediate, HPV-difficult** and **PV-non-human**– were used to benchmark and compare algorithms for speed and accuracy (Material and Methods). These data were combined with human and ‘contaminant reads’, comprising microbial and fungal reads that are often found to contaminate human samples to complete the data sets (See Material and Methods).

In addition to benchmarking test data, we also used FastViFi to identify viral reads in samples from TCGA, including DNA reads from head and neck squamous cell carcinomas (HNSC)(24) with HPV infection, RNA reads from hepatocellular carcinoma with HBV and HCV infections (HCC)(25), and RNA reads from gastric adenocarcinoma with EBV infection (STAD)(26).

Some of the HPV-difficult and all of the PV-non-human sequences were non-human PVs and represented truly difficult cases, where the reported host on PaVE was non-human. However, they are useful for benchmarking performance on highly variant strains. We note that the known cancer associated HPVs (HPV16, 18, 6,11, 31, 33, 45, 52, 58) are all Alpha PVs. These form the easiest cases, implying that the test data sets results represent a harder and more conservative measure of accuracy.

### Efficiency versus recall of two-stage versus single-stage filtering

**Figure 2.**
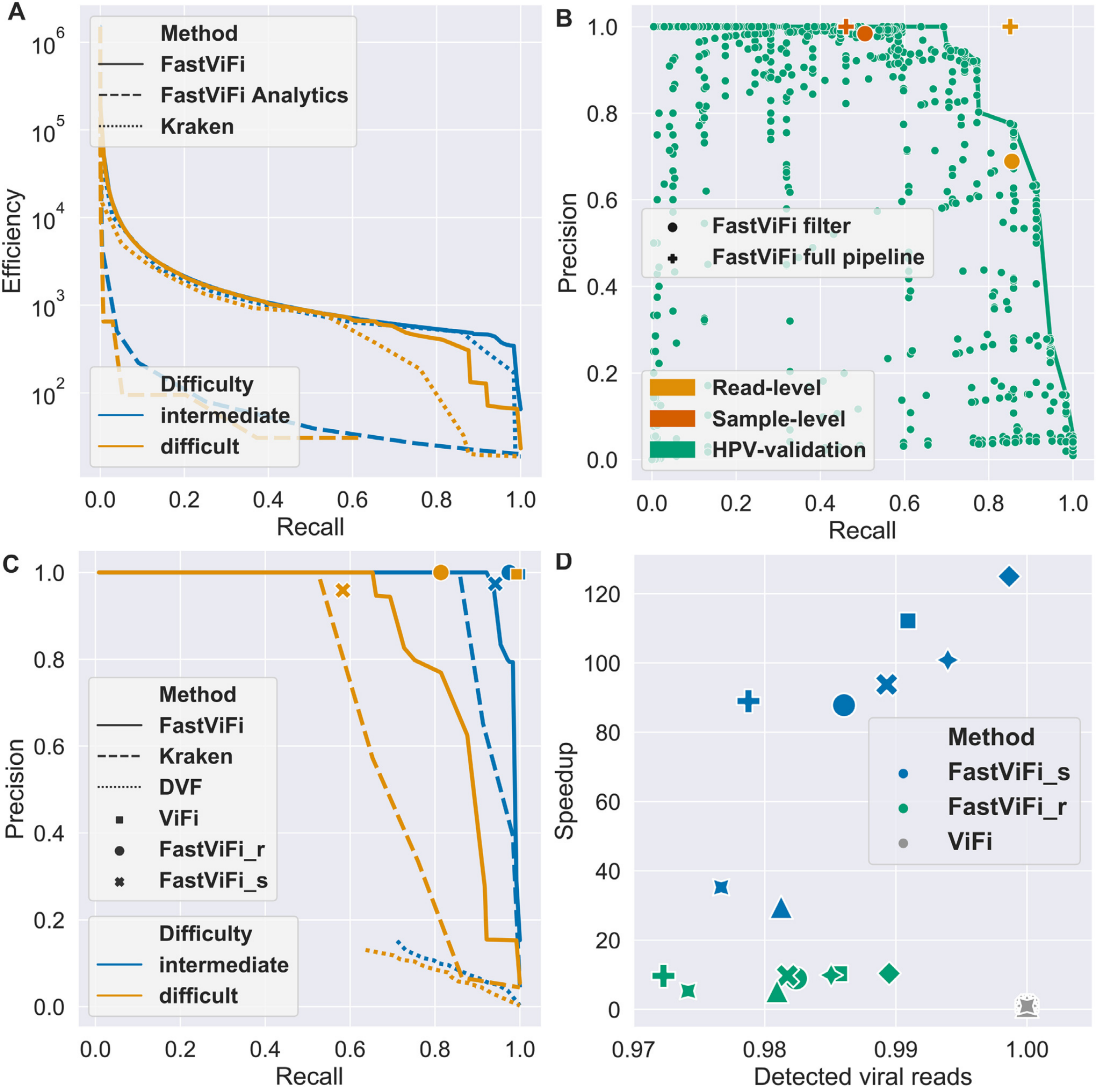
FastViFi filtering. **Panel A**. Efficiency-recall trade-offs for emprical search, FastFastViFiAnalytics predictions and single-stage Kraken on the test data sets with different parameter-settings (Also see Supplementary Figure S1). **Panel B**. FastViFi precision versus recall on HPV-validation, along with chosen settings for FastViFi_r (orange) and FastViFi_s (red). **Panel C**. Precision-recall trade-off for all the benchmarked methods. For FastViFi and Kraken, the lines were obtained by running a grid search on the input parameters and excluding the data points with suboptimal performance. For DVF, the lines are formed by varying a threshold on the score of each read to classify the read origin. **Panel D**. FastViFi speedup versus sensitivity compared to ViFi on the HNSC data set (8 samples). Each sample is represented by a dedicated marker shape. For each sample, the markers representing ViFi experiments are placed at (1.0, 1.0), and the FastViFi speed-up and sensitivity are described relative to the corresponding ViFi run.

We used the HPV-training data and the human reference to identify HPV-related and human keywords, and used a choice of the 5 parameters for a two-stage filter. We tested FastViFiAnalytics in predicting the optimal efficiency and recall for any choice of parameters (Figure 2A) against empirical results on HPV-Intermediate (blue-line) HPV-difficult (orange line) and also PV-non-human (Supplementary Figure S1, red line). The results suggest that FastViFiAnalytics can be utilized as a faster alternative to predict parameters without running a grid search.

We compared the efficiency versus recall for single-stage (labeled as Kraken) filtering versus the two-stage (labeled as FastViFi) filtering, and found that the two-stage filtering dominated single-stage (Kraken) filtering. For example, we observed a high efficiency of 432 at 94% recall in HPV-intermediate (Supplementary Figure S1B) implies that only one out of 432 reads passed the filters to the ViFi step of FastViFi on average, potentially resulting in a sharp speedup. In contrast, the single-stage Kraken filter showed an efficiency of 254 at 94% recall (Supplementary Figure S1B). This behavior was due to the single Kraken filter either permitting a larger number of non-viral reads (low efficiency) or losing true viral reads (low recall). The improved efficiency versus recall curves for the two-stage FastViFi had a direct impact on the precision versus recall curves shown later.

For all methods, there was drop in efficiency from HPV-intermediate to HPV-difficult because fewer reads could be confidently discarded by the filter without losing sensitivity.

### Training FastViFi parameters empirically

We developed the FastViFi pipeline by coupling ViFi to the output of filters with a user-defined choice of 5 parameters, and performed a grid-search (Section Material and Methods:filter parameter grid-search) on the 5 parameters, measuring precision and recall on the HPV-Validation data set (Green dots in Figure 2B) and also efficiency versus recall (Supplementary Figure S1). We chose two Pareto optimal settings. The first identified viral reads with $50\%$ recall and efficiency of 834. While the per-read recall was low in this setting, it was sufficient to test if the sample was HPV positive and provided high efficiency/speed. The second parameter achieved read-level sensitivity exceeding $80\%$ with efficiency of 392, as this setting was sufficient to identify most viral human hybrid transcripts. The pipelines corresponding to these parameter settings were denoted as as FastViFi_s (sample-level) and FastViFi_r (read-level), respectively. We compared these methods against FastViFi (Filter only with no ViFi coupling), Kraken(21) (a single level keyword filtering method optimized for HPV), DVF(18), a recent deep learning based virus finder method, and ViFi(15). VIRUSBreakend(17) requires an assembly step which failed in these sparse data-sets that are unsuitable for checking sites of integration.

### Precision versus recall

We plotted the precision versus recall trade-off across all methods (Figure 2C and Supplementary Figure S2) for HPV-intermediate and HPV-difficult. ViFi had the highest precision and recall on HPV-intermediate and HPV-difficult. On HPV-intermediate, FastViFi_r obtained recall of 0.97 with perfect precision of 1.0. In contrast, Kraken as an stand-alone tool obtained a recall of 0.86 for precision 1.0. The recall of FastViFi_r decreased to 0.81 for HPV-difficult data, which is still sufficient to identify most HPV positive samples, while the recall of Kraken decreased to 0.53 for the highest precision reached at 0.98. To establish a baseline, we also compared our results to a direct sequence alignment based method. Specifically, we ran Blastn against the training databases to identify viral reads in the test data. Blastn had a precision less than 0.1 across all test sets. The low value of precision was attributed to the large number of diverged viral reads that could only be identified by increasing the E-value cut-offs to where false sequences also matched.

Our results additionally suggest that an ideal strategy for running FastViFi on large cancer data sets would be to run FastViFi_s across the whole data set to rapidly infer viral infection, followed by running FastViFi_r on the samples deemed positive by FastViFi_s, with both options available in the GitHub repository. Across all of our test sets, DeepVirFinder achieved less than 20% precision despite retraining the models (Figure 2C). We attribute this to two key assumptions made by DeepVirFinder: (1) onco-viral genome data contain only human and viral genomes and no contaminants; and, (2) viral genomes are well-represented in the database so that the test viral genomes are adequately represented in training. The former assumption specifically, relates to *open-set* problems, with the contaminant sequences forming the unknown-unknown class (not only unseen but also having no side information during training). Open-set problems are known to be challenging for deep neural networks (27), and we observe that in our data. The second assumption does not suitably account for the high variation in viral strains.

### FastViFi rapidly and accurately identifies HPV reads in head and neck squamous cell carcinomas

We applied FastViFi to WGS data from 29 HPV positive samples from the Cancer Genome Atlas study of head and neck cancers (TCGA-HNSC). The original ViFi is very resource intensive and therefore we ran it on eight samples for comparison. For both methods, we discarded reads from the input BAM file where both mates of the paired-end read mapped to the human genome prior to analysis. Notably, FastViFi_r was 10 × faster than ViFi (Figure 2D), while retrieving (on average) $96\%$ of the reads identified by ViFi (Supplementary Table S1). FastViFi_s was 30-120 × faster with sensitivity loss of $3\%$ when compared to ViFi. The running time of FastViFi_s on the 29 HNSC whole genome samples ranged from 25 minutes to 9 hours with an average of 101 minutes per sample (Supplementary Figure S5). Most runs completed in about 2 hours. The running time was correlated with the number of unmapped reads up to 10^8^ reads, and was stable after that.

**Figure 3.**
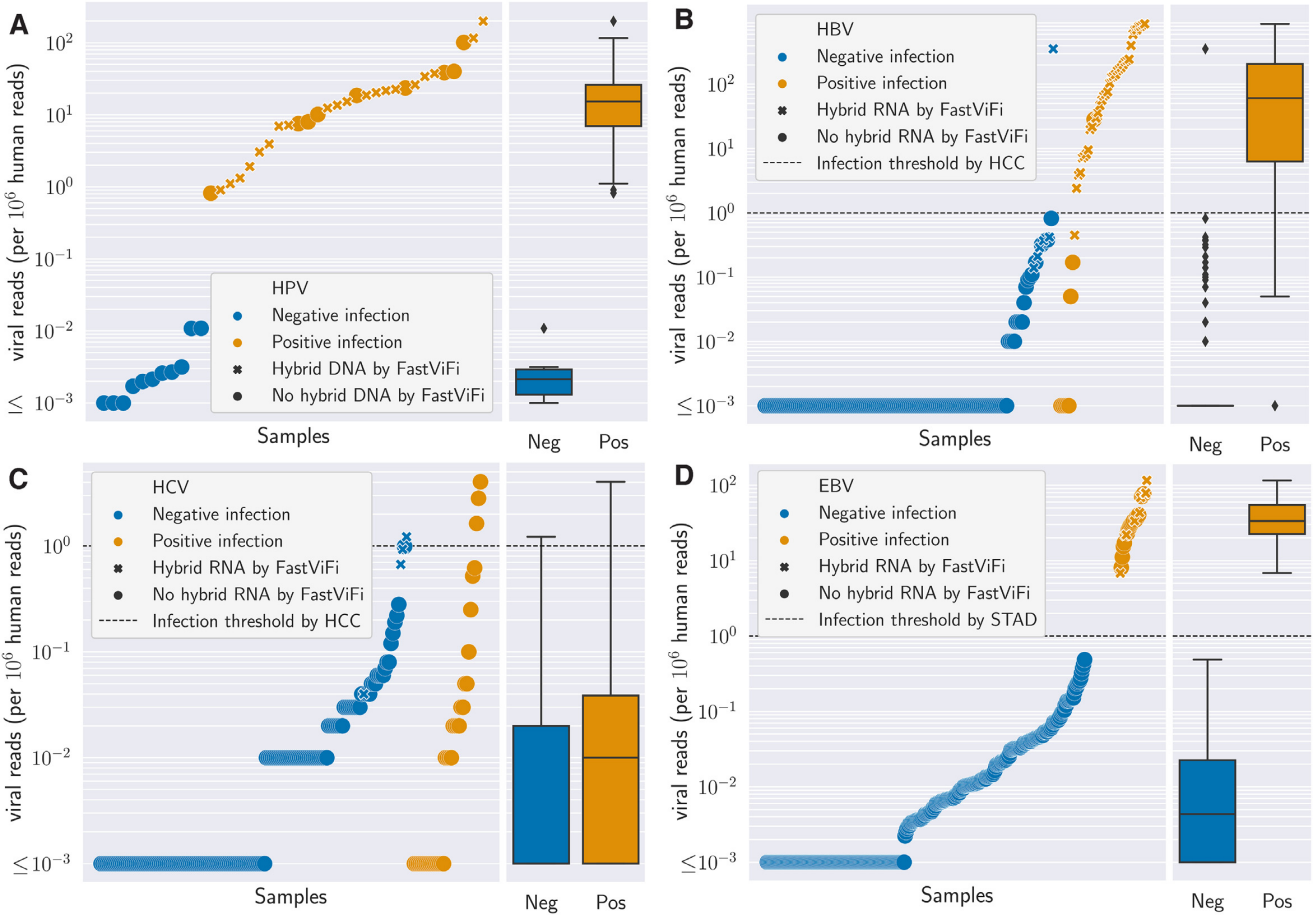
FastViFi based identification of genomic integration in HPV-HNSC (panel A), and hybrid transcripts in HBV-HCC (panel B), HCV-HCC (panel C) and EBV-STAD (panel D). The right side of each panel displays the distribution of viral read abundance for class samples labeled as infected (orange) and non-infected (blue) in the original study. Each sample is represented by one marker ordered by normalized viral reads (per 1 million human reads) as detected by FastViFi. The horizontal dashed line at y=1 across panels B,C,D is the accepted threshold for hybrid transcript detection. Each marker is a cross if hybrid transcripts (or hybrid DNA) was identified, and otherwise denoted by a circle.

In terms of accuracy on WGS data, FastViFi_s detected HPV at the threshold of ≥1 viral read per million human reads in all 29 HPV positive sequences, and in 0 of the 15 control sequences (Figure 3A). As this is a relatively ‘easy’ data set, we also tested the single stage filtering strategy. Applying Kraken as an stand alone tool could not separate the HPV negative and positive samples, and missed three HPV positive samples (sensitivity = 0.9) at the threshold of 1 viral read per million reads (Supplementary Figure S3). Moreover, in FastViFi_s we observed a significant margin between the number of viral reads (per million human reads) in HPV positive and negative classes. Such a large margin between the two classes is not observed when running Kraken. This is likely due to higher level of false positive reads reported by Kraken on HPV negative samples, compared to FastViFi_s. The results confirm that FastViFi_s can be used as a quick and accurate test for detection of HPV sequences in human samples. FastViFi_s also detected hybrid DNA in all 19 samples previously reported by ViFi.

VIRUSBreakend does not report viral-only reads but has shown excellent performance for the detection of integration locations using local assembly on simulated data (17). On 22 of HCC samples with HBV, they reported exactly the same 16 integration sites as ViFi (17). As FastViFi has a different focus, we only did a partial comparison against VIRUSBreakend using 2 WGS HNSC samples.

As recommended, GRIDSS2(28) was used for local assembly prior to running VIRUSBreakend. On one sample bam file, TCGA-CR-5243, FastViFi took 61 cpu-minutes including 37 minutes of the alignment-based filter (Section Material and Methods:pre-processing). VIRUSBreakend took 185 minutes using a single thread with most time devoted to local assembly using GRIDSS2 (173 minutes). On a second sample (TCGA-CR-6473), VIRUSBreakend took 187 minutes on a single thread (using 178 minutes for assembly), while FastViFi took 73 minutes, including 40 minutes of alignment-based filter (Section Material and Methods:pre-processing).

### FastViFi rapidly identifies viral transcripts

Expectedly, the FastViFi running time was much lower on RNA-seq data for HCC and STAD, with a mean time of 2 minutes for HCC and 9 minutes for STAD (Supplementary Figure S5). Notably the Epstein Barr Virus (EBV) is significantly larger than HBV and HPV (170kbp vs. 3kbp and 7kbp), which translates to slower HMM alignments. Nevertheless, the running time on STAD RNA-seq samples was well below DNA samples in HNSC.

We could not match these results on the RNA-seq samples using VIRUSBreakend, which is not configured for RNA. On a single sample of aligned reads (Barcode TCGA-FP-7998-01), the FastViFi pipeline took 40 minutes including 27 minutes of the alignment-based filter (pre-processing, see Material and Methods) using a single CPU-thread on a server with 126Gb RAM and 24 CPU-threads. VIRUSBreakend (with 60Gb heap allocation) ran for 6 days on the server but failed at the assembly step. Therefore, we did not run it on all samples.

### Hybrid viral-human transcripts are abundant in liver and gastric cancers

We applied FastViFi to a comprehensive RNA-seq data set with 193 Hepatocellular carcinoma (HCC) cases from the Cancer Genome Atlas(25). In that study, three separate computational methods as well as clinical markers were used to determine the HBV/HCV status of the patients, resulting in 44 tumors being labeled as HBV positive, and 31 as HCV positive. These positive samples included cases where clinical evidence confirmed viral infection, but none of the computational models reported viral infection possibly due to a response to antiviral therapy, among other reasons.

FastViFi identified at least 1 viral read per 1 million human reads in 36 (82%) of the HBV positive samples and in 1 HBV negative sample (Figure 3B). In contrast, HCV transcript abundance was much lower. In the HCC cohort, only 3 of the 35 HCV positive samples and 1 of the 161 HCV negative samples met the required threshold for detection (Figure 3C) Similar to HBV, FastViFi identified a high burden of EBV transcripts in all of the 27 samples that were reported as EBV positive and none of the EBV negative samples that had RNA-seq data available in the original study(26) (Figure 3D).

Overall, our results suggest that viral HBV and EBV transcript abundance values strongly correlate with infection status at diagnosis, and point to the applicability of FastViFi in transcript based detection of HBV mediated liver cancers and EBV mediated gastric cancers.

#### Viral-human hybrid RNA

Out of the 37 samples with HBV-human hybrid RNA reported by the HCC study, FastViFi detected HBV-human hybrid transcripts in 36, failing to report hybrid RNA for only one sample while still labeling the sample as HBV positive (Supplementary Table S3). Additionally, FastViFi identified viral-human hybrid in one HBV(-) and also one HBV(+) sample where the hybrid RNA were not detected in the HCC study (Supplementary Tables S4, S5). Similarly, it reported hybrid RNA in 4 HCV(-) samples not previously reported as carrying hybrid RNA in the TCGA study(29). Only one of these samples met the threshold of 1 viral read per 1 million human RNA reads. Notably, FastViFi identified strong evidence of HBV-human hybrid RNA in one sample (TCGA-CC-A1HT-01A) which was labeled as HBV(+) in the HCC study, but no hybrid RNA was reported (Supplementary Table S4 and Supplementary Figures S6, S7).

We additionally tested FastViFi for detecting EBV from 371 of 440 samples in the gastric cancer (TCGA-STAD) cohort(26) where RNA-seq was available. EBV integration into the host genome was generally considered to be infrequent in gastric cancer(26,29,30). However, a recent study identified multiple genomic integration sites through a targeted sequencing approach(31). The TCGA study(26) had previously confirmed a single EBV-human hybrid transcript through long-read sequencing. FastViFi confirmed that transcript identified in the STAD study (barcode TCGA-FP-7998), but also reported hybrid EBV-human RNA in 9 additional samples representing 38% of EBV positive gastric cancers in the TCGA-STAD cohort (Figure 3D and Supplementary Table S6). Together, our data suggest that similar to HPV(15), HBV and EBV mediated cancers frequently carry viral-human hybrid transcripts.

### HBV and EBV hybrid transcripts mediate tumor development

**Figure 4.**
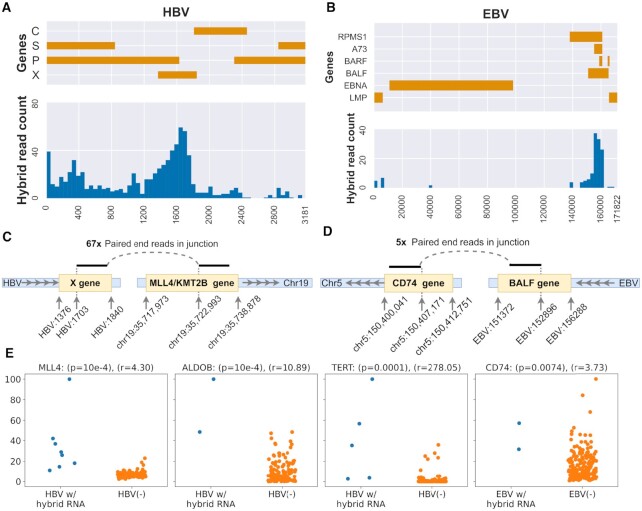
Hybrid human-viral RNA loci. Panels A, B. The two histograms show the location of the integration sites on HBV and EBV, respectively. The integration locis is non-random, enriching for HBx gene on HBV (panel A) and the BALF gene cluster on EBV(panel B) EBV. **Panels C, D**. Illustration of hybrid transcripts for HBx (NC_003977.2) and EBV(NC_007605.1) fused to MLL4 and BALF5 respectively (GrCH38 coordinates). One paired end read was chosen to represent each junction. **Panel E**. Normalized abundance of hybrid isoforms relative to their non-hybrid isoforms. ‘r’ denotes the ratio of expression means in the two sets. For ease of exposition, the RSEM values were further normalized by the maximum expression of the gene in each panel. A permutation test on ranks was used to compute p-values(Material and Methods).

In the HCC cohort, we identified 793 hybrid transcript junctions after clustering junctions within 100bp (Material and Methods). On the viral genome, $337 (42\%)$ of these 793 junctions originated at the HBx gene (Figure 4A). The product of HBx is an oncogenic marker known to interact with many other genes including TP53 and TERT to promote oncogenesis in liver cancer(32). While most hybrid reads matched directly to an HBV reference, 46 individual reads were discovered through a mapping to HBV HMMs. Interestingly, these 46 reads all mapped to an HBV locus containing the S and P genes in overlapping frames (Figure 4A). These results suggest that HBV-S hybrid transcripts are prevalent in a non-reference strain of HBV. Integration at the HBV-S gene locus is incompletely understood. However, integration of the ESPL-1 gene with HBV-S is associated with hepatocellular carcinoma in Chinese populations(33).

The HBV hybrid junctions involved 91 distinct human genes (Supplementary Table S7) of which 27 (30%) were cancer associated, in contrast with the 11% of all genes that are cancer associated (P-value: 0.0001; χ^2^-test). ancer associated genes were derived from the allOnco gene list accessible through http://www.bushmanlab.org/links/genelists (2018). Many genes appeared recurrently including KMT2B/MLL4 (10 times), TERT (8), SERPINA1 (8) (Supplementary Table S8). Our results supported previous publications that reported hybrid TERT and KMT2B transcripts in HCC cell-lines(34).

A gene-set enrichment analysis using DAVID(35) showed enrichment for blood microparticles (BH q-value: 7.41 · 10^−8^) and platelet α-granule lumen proteins (q-value: 1.73 · 10^−7^). Adhesive proteins found in α-granules mediate direct interactions between tumors and platelets and platelet adhesion may facilitate tumor metastasis through cloaking tumor cells from immune surveillance and assisting their egress from circulation(36).

Similarly, FastViFi identified 141 junctions in EBV associated STAD samples (Supplementary Table S9). Four of these junctions involved EBV HMM matches while the rest matched the reference EBV sequences. On the EBV genome, the junctions enriched (Figure 4B) for a region contained eight genes (BARF0, BALF3, BALF4, BALF5, A73, RPMS1, LF2 and LF1) that are highly expressed in the majority of EBV-positive gastric cancers(37). The BALF family of genes are involved in the early lytic phase of the EBV life-cycle(38).

The EBV-hybrid transcripts included 74 human genes, with B2M(2), CD74(2) and ACTB(2) occurring recurrently (Supplementary Table S10). Eighteen (24%) of the 74 genes were cancer associated (p-val: 0.0005). Enrichment analysis using DAVID(35) revealed that 32 of the 74 genes encoded components of the extracellular exosome cellular component (68 genes; GO:0070062; BH q-value:3.8 · 10^−7^). Extracellular vesicles (EVs) have been previously implicated in EBV pathogenesis(39), and this enrichment suggests a novel role of EBV hybrid transcripts in extracellular vesicle formation. In thirty of the 91 transcripts integrated with HBV and 30 of the 74 genes integrated with EBV, the overlap was with coding exons (Figure 4C,D) consistent with viral promoters driving the expression of the viral-human hybrid transcript. We tested the impact of this integration on gene expression relative to the expression of the same gene in non-integrated samples. In many cases, the hybrid transcripts were significantly over-expressed for both HBV and EBV with the mean expression of the gene 4 - 278x higher in a hybrid isoform relative to the non-hybrid isoform. (Figure 4E).

## DISCUSSION

There is tremendous diversity in viral sequences even within a single family. Accurate detection of viral sequences in a mix of host and contaminant genomes are not currently achieved by discriminative deep learning models, even ones devised and trained specifically for this purpose. Representational models such as Hidden Markov models show high sensitivity but only when an ensemble of models is used - each capturing the diversity in a single clade. This leads to a computational bottleneck, especially when searching large data sets of host genomes for presence, absence or integration of the virus. Our results suggest that a 2-stage filtering is effective and fast, with the first stage used largely to eliminate the bulk of the host sequences, and the second filter used mainly to retain the viral sequences while discarding other contaminants. The small number of filtered reads can then be searched with the HMM ensemble to identify viral reads. FastViFi can search typical human whole genome data sets in an average time of 1 hour and RNA-seq data in <10 minutes.

Methods such as VIRUSBreakend are excellent for detecting locations of viral integration into the host genome, but require a prior assembly step to ensure extension of repetitive sequence into unique regions. The GRIDSS2 assembly is necessary for VIRUSBreakend, but may not be a standard part of the pipeline of other tools, and therefore running VIRUSBreakend could add significant computational expense.

We designed FastViFi for a quick identification of viral reads in WGS, and viral and hybrid transcripts in RNA-seq data. We ran FastViFi on different oncoviral data sets including HNSC whole genomes and RNA-seq for HBV, HCV and EBV. The results suggested interesting differences in viral pathology mechanisms between HPV versus HBV and EBV. HPV integration in the human host occurs without preference for particular loci or genes. They act primarily to promote production of viral oncogenes E5/E6. HBV and EBV showed clear preference for the location of the viral end at HBx, HBV-S, and EBV-BALF regions. Also, the human part was often located in a protein coding gene, sometimes recurrently. The hybrid transcripts were consistent with a translated product supporting the viral gene fused to a human coding gene and the viral promoters driving transcription of the fused human genes.

Previous reports have suggested increased pathogenicity in HBV hybrid transcripts due to activation of LINE elements(34). Our results point to a role of human oncogenes in mediating this pathogenicity. Other reports suggest increased genome instability due to HBV integration(40). We plan to investigate these in future work. Similarly, integration of EBV in gastric cancers has been associated with global changes in gene expression(41), and it will be intriguing to test if some of these hybrid transcripts drive those changes. In conclusion, FastViFi will add to the toolkit available to scientists studying viral infections and hybrid viral-host transcripts.

## Supplementary Material

lqac032_Supplemental_Files
