# Supplementary material for: FastViFi: Fast and accurate detection of (Hybrid) Viral DNA and RNA
Source: NAR Genom Bioinform. 2022 Apr 26;4(2):lqac032. doi: 10.1093/nargab/lqac032 (PMC9041341; doi:10.1093/nargab/lqac032)
Supplement: lqac032_Supplemental_Files [file lqac032_supplemental_files.zip › FastVifi_supplementary_figures.pdf]

SUPPLEMENTARY SECTION

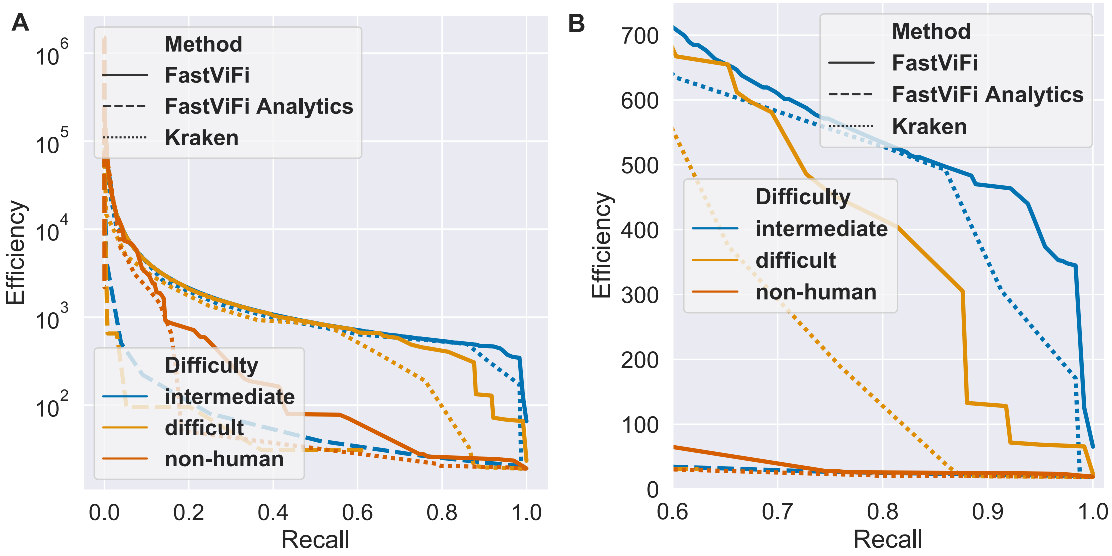

Figure S1. Efficiency-recall plot for all the filters used in this study, namely FastViFi filter (no ViFi step), FastViFi Analytics and Kraken (single kraken filter) on the intermediate, difficult and non-human test datasets. **Panel A** shows the full range of recall and **Panel B** shows the zoomed-in region on recall >0.6. FastViFi filter outperforms a single Kraken filter with human and viral reference indices in all test datasets. FastViFi Analytics closely replicates FastViFi filter performance, especially in the intermediate test data set. Finally, all methods have reduced efficiency as the level of difficulty increases in the test data sets.

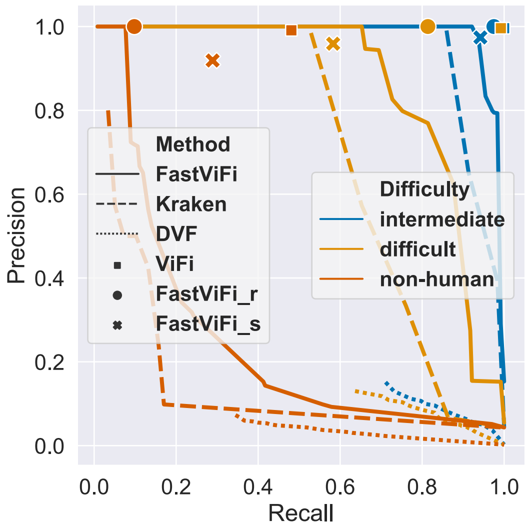

Figure S2. Precision-recall plot for all the methods discussed in this study on the test data sets: intermediate, difficult and non-human. FastViFi denotes the FastViFi filters with no ViFi step. FastViFi_r and FastViFi_s denote the read-level and sample-level FastViFi (with ViFi step) respectively. Single Kraken filter (with human and viral references) is denoted by Kraken. Finally, DVF and ViFi represent the DeepVirFinder and ViFi tools.

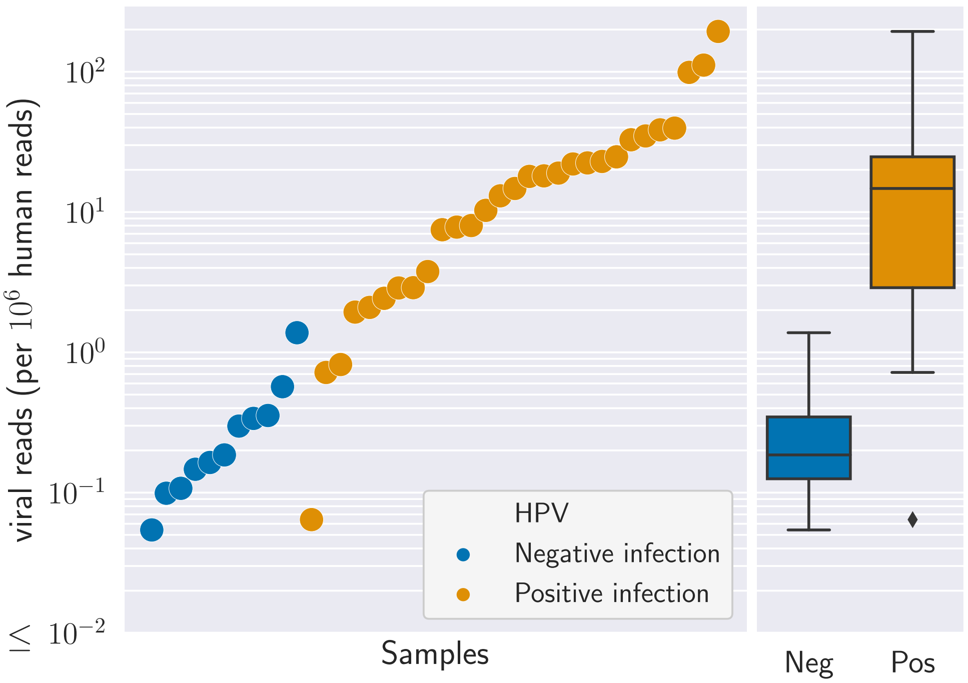

Figure S3. Classification of HPV infection by Kraken2 as an stand-alone tool on the HPV-HNSC samples. The left side of the figure shows the number of viral reads (per 1 million human reads) found by Kraken in each sample. Samples are colored based on the labeling in the original study: Orange as positive and blue as negative. The right side of the figure displays the distribution of viral reads per infection class. Notice the log scale on both panels.

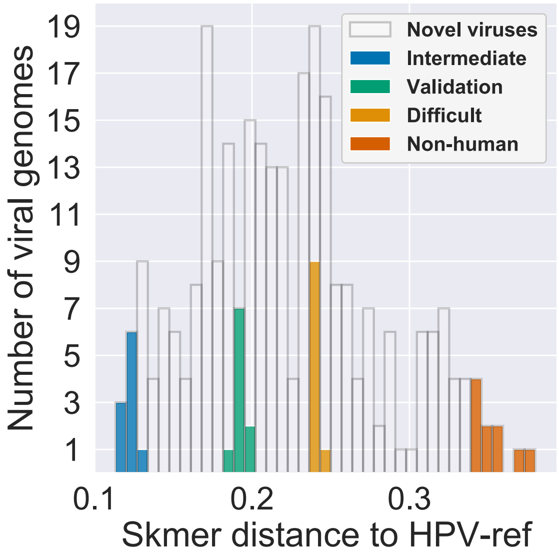

Figure S4. Skmer distance to HPV-ref computed with k = 15. Easy and difficult test set were chosen from the viruses with smallest and largest Skmer distance to the closest viral genome in the HPV-ref respectively with mean values of 0.12 and 0.35 respectively. The validation and intermediate data sets were chosen based on the 33rd and 66th percentile of Skmer distances to closest viral genome in HPV-ref respectively with mean values of 0.19 and 0.24 respectively.

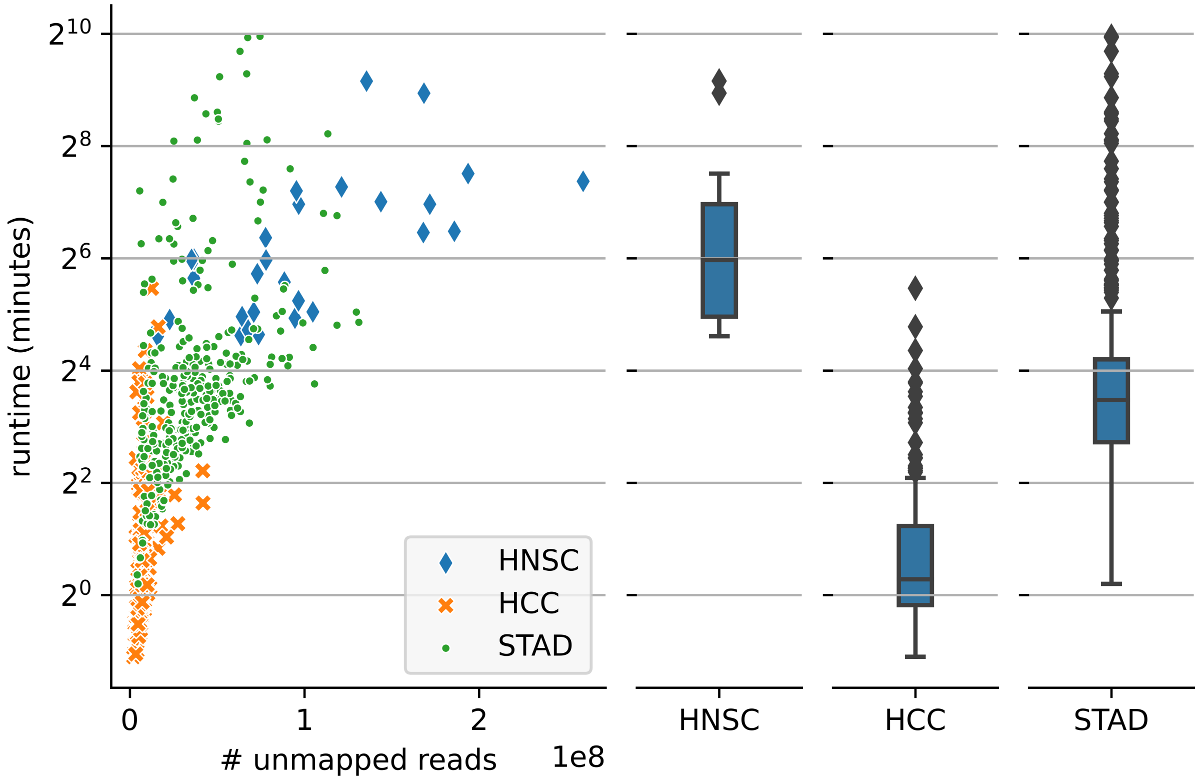

Figure S5. Running time for running sample-level FastViFi on a single thread on the three studies HNSC, HCC and STAD corresponding to viruses HPV, HBV (and HCV), and EBV respectively.

```
QUERY: UNC12-SN629_127:4:1206:14079:61441

SCORE START   END QSIZE IDENTITY  CHROM  STRAND  START       END   SPAN
---------------------------------------------------------------------------
   50     1    50    50   100.0%  chr5    +     1294599   1294648     50


Query 0000001 ACGTTCTTCGCGCCGCGCTCGCACAGCCTCTGCAGCACTCGGGCCACCAG 0000050
              ||||||||||||||||||||||||||||||||||||||||||||||||||
Chr5  1294599 ACGTTCTTCGCGCCGCGCTCGCACAGCCTCTGCAGCACTCGGGCCACCAG 1294648
```

A. One mate mapped to the human genome chr5:1294599-1294648 (GRCh38), Exon 2 in TERT gene.

```
QUERY: UNC12-SN629_127:4:1206:14079:61441

>Hepatitis B virus isolate 1074_4w_C39, partial genome
Sequence ID: MT427047.1 Length: 3215
Range 1: 299 to 348

Identities:49/50(98%),  Gaps:0/50(0%), Strand: Plus/Plus

Query     1    CTTGGCCAAAATTCGCAGTCCCAAATCTCCAGTCACTCACTAACTTGTTG  50
               |||||||||||||||||||||||||||||||||||||||| |||||||||
Sbjct     299  CTTGGCCAAAATTCGCAGTCCCAAATCTCCAGTCACTCACCAACTTGTTG  348
```

B. The other mate mapped to the HBV genome

Figure S6. Alignment of a representative hybrid HBV-human read (read id: UNC12-SN629_127:4:1206:14079:61441) for sample with the barcode TCGA-CC-A1HT-01A on the HCC cohort. Alignments and locations were confirmed using NCBI BLAST and UCSC genome browser BLAT.

```
QUERY: UNC12-SN629_127:4:1102:18534:2127

SCORE START   END QSIZE IDENTITY  CHROM  STRAND  START      END   SPAN
-----------------------------------------------------------------------
   50     1    50    50   100.0%  chr5   +      1294604  1294653    50


Query 0000001 CTTCGCGCCGCGCTCGCACAGCCTCTGCAGCACTCGGGCCACCAGCTCCT 0000050
              ||||||||||||||||||||||||||||||||||||||||||||||||||
Chr5  1294604 CTTCGCGCCGCGCTCGCACAGCCTCTGCAGCACTCGGGCCACCAGCTCCT 1294653
```

A. One mate mapped to the human genome chr5:1294604-1294653 (GRCh38), Exon 2 in TERT gene.

```
QUERY: UNC12-SN629_127:4:1102:18534:2127

>Hepatitis B virus isolate 1074_4w_C39, partial genome
Sequence ID: MT427047.1 Length: 3215
Range 1: 372 to 421

Identities:50/50(100%),  Gaps:0/50(0%), Strand: Plus/Plus

Query     1    GCTGGATGTGTCTGCGGCGTTTTATCATCTTCCTCTGCATCCTGCTGCTA  50
               ||||||||||||||||||||||||||||||||||||||||||||||||||
Sbjct     372  GCTGGATGTGTCTGCGGCGTTTTATCATCTTCCTCTGCATCCTGCTGCTA  421
```

B. The other mate mapped to the HBV genome

Figure S7. Alignment of a representative hybrid HBV-human read (read id: UNC12-SN629_127:4:1102:18534:2127) for sample with the barcode TCGA-CC-A1HT-01A on the HCC cohort. Alignments and locations were confirmed using NCBI BLAST and UCSC genome browser BLAT.
